# Microfiber Pollution: A Systematic Literature Review to Overcome the Complexities in Knit Design to Create Solutions for Knit Fabrics

**DOI:** 10.1021/acs.est.3c05955

**Published:** 2024-02-21

**Authors:** Elisabeth Allen, Claudia E Henninger, Arthur Garforth, Edidiong Asuquo

**Affiliations:** Department of Materials & Engineering, University of Manchester, Oxford Road, M13 9PL Manchester, United Kingdom

**Keywords:** microfiber pollution, textile design, knit, systematic literature review, microplastic fibers

## Abstract

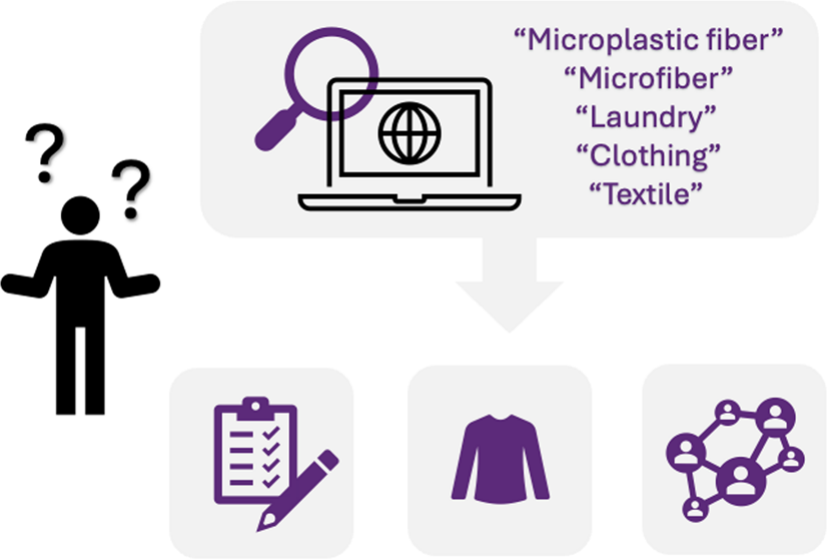

The absence of standardized procedures to assess microfiber pollution released during laundering, alongside textile complexities, has caused incomparability and inconsistency between published methodologies, data formats, and presentation of findings. Yet, this information needs to be clear and succinct to engage producers and consumers in reducing microfiber pollution through solutions, such as eco-design. This review analyses source directed interventions through design and manufacturing parameters that can prevent or reduce microfiber shedding from knit fabrics during washing. Contradicting results are critically evaluated and future research agendas, alongside potential areas for voluntary and involuntary sustainable incentives are summarized. To do this, a systematic review was carried out, using the PRISMA approach to verify which fabrics had been investigated in terms of microfiber shedding. Using selected keywords, a total number of 32 articles were included in this review after applying carefully developed inclusion and exclusion criteria. The influence of fabric parameters such as fiber polymer, length of fibers and yarn twist alongside fabric construction parameters such as gauge of knit and knit structure are critically evaluated within the systematically selected studies. This review highlights the agreed upon fabric parameters and constructions that can be implemented to reduce microfiber pollution released from knit textiles. The complexities and inconsistencies within the findings are streamlined to highlight the necessary future research agendas. This information is critical to facilitate the adoption of cross-industry collaboration to achieve pollution reduction strategies and policies. We call for more systematic studies to assess the relationship between individual textile parameters and their influence on microfiber shedding. Additionally, studies should work toward standardization to increase comparability between studies and created more comprehensive guidelines for policy development and voluntary actions for the textile and apparel industry to participate in addressing more sustainable practises through eco-design.

## Introduction

Microfiber pollution is a key priority area that has gained increased attention, with research estimating “over 14 million tonnes of [microfibers] have accumulated on the world’s ocean floor” and a further 200–500,000 tonnes of microplastic fibers are entering the ocean annually.^1^ It must be noted that microfibers released via the textile and apparel industry can be created from a variety of polymers including natural (e.g., cotton, wool), synthetic (man-made from an artificial product, e.g., polyester, polyamide) and semisynthetic (man-made from a natural product, e.g., rayon, acetate).^2,3^ Therefore, the term microfibers is used to encompass natural, synthetic, or semisynthetic fibers of less than or equivalent to 5 mm in length.^2^ Currently, most research focuses on synthetic microfibers and excludes natural fiber pollution even though this neglects “a major component of anthropogenic microfiber pollution”.^4^

As much of the research is focused on microfibers from synthetic sources, the term microfiber has historically often been used interchangeably with microplastics, however distinctions need to be made based on the raw material of the microfibers.^5−7^ As this research area evolves, alternative phrases such as “fiber fragments” have been suggested to avoid ambiguity, as microfibers can also be used to describe fibers with a certain diameter and denier as well as a type of brushed/processed fabric commonly called fleece.^8,9^ However, the use of fiber fragments is not widespread yet due to its relatively recent emergence and therefore this systematic literature review (SLR) will continue to use “microfiber”, which here also includes “fiber fragments”.

**Figure 1:**
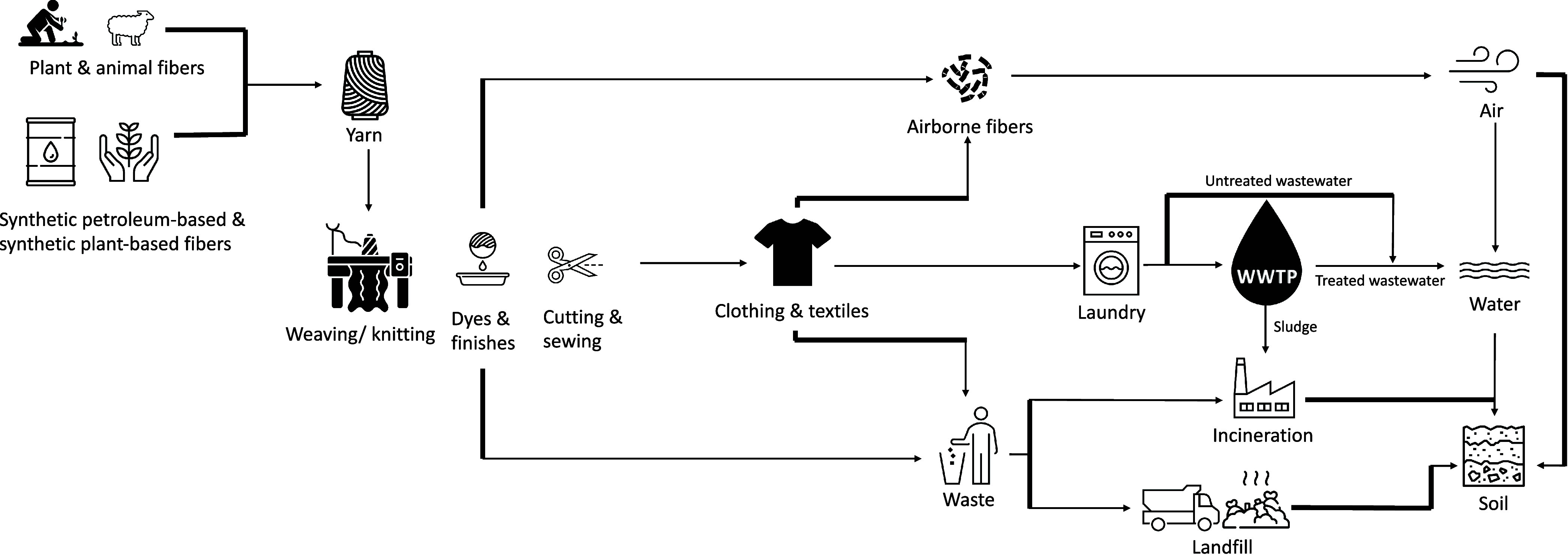
Diagram of microfiber shedding from clothing throughout the textile material through its life cycle including production, usage, and disposal. (Authors’ own representation).

Within the textile and apparel context, microfibers can be released or broken off from garments structures throughout a garment’s lifetime, including production, use, and end-of-life. These fibers can either be airborne and break off garments/textiles in use, or during the laundry/cleaning process and are thus waterborne as seen in Figure 1.^8^ Of particular importance, waterborne microfibers released during the washing of textiles and apparel have been highlighted as significant pollutants to our marine and terrestrial environment, with fibers being found in deep sea sediment and some of the most remote locations on land (e.g., Himalayas).^10,11^ Napper and Thompson^12^ estimated that 6 kg of clothing releases over 700,000 microfibers during a single laundry cycle, with other sources showing further variations in this number, to either be higher and/or lower in value depending on the garment’s fabric.^12−14^

In 2017, it was estimated that washing of synthetic garments and the subsequent microplastic fibers released were the largest contributor to microplastic pollution in our oceans.^15^ Furthermore, as the textile industry is “regarded as one of the most chemical-intensive industries on the planet” it is unsurprising that the release of microscopic fibers is an environmental concern.^16^ Once in the marine and terrestrial environment, microfibers can adsorb heavy metals and other pollutants and act as vectors of chemicals within their polymer structure or adsorbed onto their surface to other environments and into living organisms.^17−19^ This has been shown to lead to altered immune systems, growth inhibition, physical injuries, and fecundity alterations.^17−19^ While the full extent of the impacts of microfibers remain unknown “irrespective of marine, freshwater, or soil ecosystems, evidence indicates that microfibers have substantially adverse effects and can enter the food chain, ultimately posing a great risk to human being”.^17^

Over the past decade research has pressed to identify sources of microfibers, the amount of pollution released into the environment, and the fate of microfibers.^20−23^ To date, microfiber pollution and research surrounding it has increased substantially in importance with microfiber pollution having been made a key priority within the recently published EU circular economy action plan.^1,24^

Although microfiber pollution has received increased attention, thus far, little research has addressed upstream solutions to reduce the amount of microfibers that are reaching the environment. This implies that there is a lack of clear yarn and fabric parameters that the textile and apparel industries can incorporate to reduce microfibers that are shed during a textile’s lifetime.^25^

The main solutions to reduce microfiber pollution currently available to consumers include end-of-pipe interventions, such as filters on washing machines.^26^ However, these solutions are rather complex and not only require incorporation of technology into washing machines or retrofitted onto the wastewater pipe but also consumer commitment.^27^ While filters may be one solution, currently they lack standardization and/or a certificate that outlines their effectiveness. To reiterate a previous point further, filters also require consumer engagement and cooperation in that consumers need to clean filters and ensure that microfibers are disposed of “correctly”, which currently implies landfilled rather than washed down the drain.^28,29^ Other solutions highlighted in the industry are objects that can be used in washing machine drums, yet, this provides challenges in terms of increased friction and thus, more shedding, or increased water use in order to ensure that washing powder residue is removed from garments. This begs the question of whether prevention strategies should be source directed, thereby removing responsibility from consumers and other stakeholders and puts responsibility onto the textile and apparel industry.

While all mitigation strategies should work in synergy, it is currently unclear which ones should be combined and how. Currently one of the biggest challenges is the overreliance on single stream solutions (e.g., filtration systems). Thus, future initiatives should drive to prevent and reduce microfiber pollution released through source-directed interventions such as policies, legislations, and eco-design of textiles and apparel as outlined by the European Commission.^24^ Eco-design here refers to actions to limit microfiber release and pollution from clothing during product manufacturing, customer use, and end-of-life. Eco-deign measures have been suggested to be enforced via extended producer responsibility policies.^30^ This is in line with the Ellen MacArthur Foundation, in that the “focus needs to be placed on the design and production stages in order to avoid fiber fragmentation and, therefore, the potential for microfiber release in the first place”.^31^

Upcoming regulations commissioned by the European Union will make the textile and apparel industry take greater responsibility for the environmental impacts of the clothes they produce through extended producer responsibility, and taxations or VAT reduction, in favor of reducing microfiber pollution.^24,30^ Furthermore, as key stakeholders strive for the industry to align strategies to meet the UN Sustainable Development Goals and market their products toward the “true cost across environmental and social factors” it is advantageous for companies to support more sustainable practises, including designing out microfiber shedding.^32^

This review analyses source directed interventions through design and manufacturing parameters that can prevent or reduce microfiber shedding from knit fabrics during washing. Contradicting results are critically evaluated and future research agendas, alongside potential areas for voluntary and involuntary sustainable incentives are summarized.

## Methodology and Material Collection

**Figure 2:**
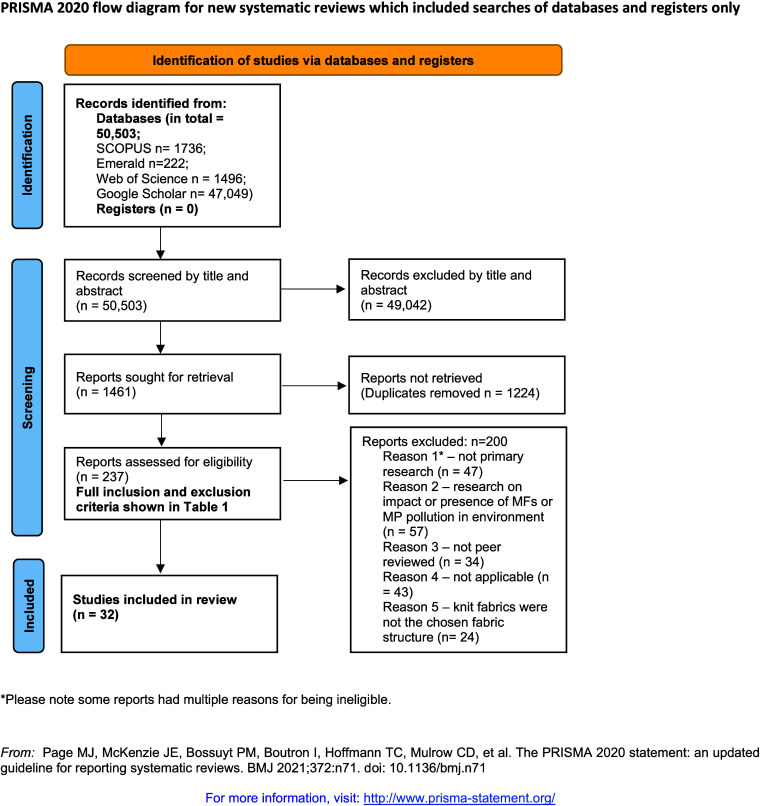
PRISMA 2020 flowchart diagram. Adapted from Page et al.^33^

This SLR utilized the Preferred Reporting Items for Systematic Reviews and Meta-Analyses (PRISMA) 2020 statement which allows authors to “transparently report why the review was done, what the authors did, and what they found”.^33^ The PRISMA 2020 statement (from here on PRISMA) consists of a detailed 27-item checklist that is worked through over four phases, and thus can be replicated by others wishing to conduct the same research.^33^ Moreover, PRISMA is a commonly used tool and has been previously utilized in SLRs that also focus on a highly complex and topical areas (e.g., Dissanayake and Pal; Jagaba et al.).^34,35^ The PRISMA 2020 checklist is commonly accompanied by a flowchart diagram, visualized in Figure 2, which outlines the individual steps taken to reach the final number of articles analyzed. These are further detailed in the following sections.

A keyword search was conducted in four databases (Web of Science, SCOPUS, Google Scholar and Emerald) to investigate the current knowledge and methodologies used to assess microfiber shedding during washing cycles, specific to knit fabrics. These databases were chosen as they gave a wide range and scope of search and had previously been used in similar studies.^4,21,36^

Knit fabrics have been chosen as these are common structures to produce fast fashion items, including, but not limited to t-shirts, jumpers, or socks. Knit fabrics were also reviewed in isolation, as knit fabrics typically release more microfibers than woven fabrics due to structural compactness.^37,38^ Thus, stopping the release of microfibers from knit fabrics is of high concern. Additionally, within commercial use, knit fabrics typically have more parameters such as yarn hairiness, twist, and fabric structure altered for design and aesthetic reasons compared to woven fabrics which are typically used for durable outerwear. It is important to note that microfibers shed from woven fabrics should not be disregarded, and this is an area of future research.

Key search terms were “microplastic fiber” and “microfiber”, along with three keywords “laundry”, “clothing”, and “textile”. Since 2004, “microplastic” has been used when describing “microscopic plastic fragments and fibers”.^3^ To ensure this SLR encapsules studies investigating the release of fibers of knit clothing and textiles “microplastic fiber” was used. Browne et al. were the first to apply the term microfiber to the micropollutant, which assesses microfibers created from synthetic polymers and natural sources and thus “microfibre” was used within the searches.^2^ The differential spelling of fibre in the UK and fiber in the US has meant both “microfibre” and “microfiber”, “microplastic fibre” and “microplastic fiber” were used within our search. “Fiber fragment” was omitted as a search term as it was a term still in its infancy and the use of “fiber” within the other search terms would have allowed relevant articles to be included.

The secondary terms were “laundry”, “clothing”, and “textile”. Laundry was chosen as it encapsules the act of washing clothes and textiles. Preliminary searches showed laundry was a common word used in the title of research papers of interest.^39−44^ “Textile” and “clothing” were chosen as they are interchangeable to assess the microfiber shedding rates from materials and were used within trailblazing studies that first discussed microfiber pollution.^2,3^ Both keywords were chosen to assess pollution from full garments (clothing) as well as fabric swatches (textiles).

The search period was between January to April 2023, with the time frame including articles from 2004 to 2023 due to Thompson et al. publishing a paper on findings of microplastic fibers.^3^ The screening criteria was conducted in stages and is summarized in Figure 2. Initial searches produced 50,503 literature references. The titles and abstracts were initially screened using the inclusion and exclusion criteria (Table 1), which form a vital part of PRISMA’s 27 item check list.^33^ From the initial search, 49,042 potential literature references did not meet the inclusion criteria, and after a removal of 1224 duplicate papers, the remaining 237 publications were evaluated and 32 fully met the inclusion criteria. Additional research papers were added within the finding’s sections, although they were not part of the SLR. This has been consciously done, to support and further back up the conclusions that were drawn.

**Table 1:** Summary of the Inclusion and Exclusion Criteria for Articles Included in the SLR

Inclusion	Exclusion
Peer reviewed primary research article.	The record being a review article, conference paper, academic theses, report or from a book chapter.
The main body of the record is in English.	The main body of the record was not in English.
Published from 2004 to April 2023.	The study did not investigate knit fabrics.
Available from SCOPUS, Google Scholar, Emerald, and Web of Science.	The study investigated the impact of microfibers or microplastic pollution
The study investigated quantities of microfibers shed during laundering of fabric swatches or garments.	The study investigated the presence of microfibers in organisms or environment.
Sufficient information provided in all these areas: fabric used, washing parameters, washing equipment and microfiber recovery/isolation, identification, and quantification methods.	The study investigated airborne emissions or tumble driers.
	The study did not provide sufficient evidence in all these areas: fabric used, washing parameters, washing equipment and microfiber recovery/isolation, identification, and quantification methods.

## Results

Within this SLR 32 publications were analyzed, which are summarized in Table 2. Overall, three main areas of research were identified, in terms of how:

(1) Washing parameters impact the emissions of microfibers from knit fabrics (i.e., Cesa et al.; Dalla Fontana et al.; De Falco et al.; Hazlehurst et al.; Hernandez et al.; Kelly et al.; Lant et al.; Rathinamoorthy and Raja Balasaraswathi; Napper and Thompson; De Falco et al.; Volgare et al.; Cotton et al.; Jiang et al.; Choi et al.)^12,41−43,45−54^
(2) Efficient microfiber traps can remove microfibers from laundry wastewater (i.e., Kärkkäinen and Sillanpää; Browne et al.; De Falco et al.; Napper et al.)^55−58^
(3) Fabric parameters of knit fabrics influence microfiber shedding (i.e., Belzagui et al.; Cesa et al.; Zambrano et al.; Carney Almroth et al.; Hazlehurst et al.; Rathinamoorthy and Raja Balasaraswathi; Rathinamoorthy and Raja Balasaraswathi; De Falco et al.; Hernandez et al.; Vassilenko et al.; Özkan and Gündogdu; Raja Balasaraswathi and Rathinamoorthy; Napper and Thompson; Zambrano et al.; Dalla Fontana et al.; Frost et al.; Cai et al.; Choi et al.)^12−14,38,44,45,48,49,54,59−67^
(4) Standardization of test method for microfiber shedding from textiles (i.e., Hazlehurst et al.; Jönsson et al.)^48,68^

**Table 2:** Journals Where Final Selected Articles Were Published and their Key Aims of Research

Journal	Category	Subdiscipline	Number of Studies	Publications (reference)	Key Aim of Research
*Environmental Pollution*	Environmental Science	Health, Pollution	5	Belzagui et al., 2019^59^	Fabric parameters
				Cesa et al., 2020^45^	Washing parameters/fabric parameters
				Dalla Fontana et al., 2020^46^	Washing parameters
				De Falco et al., 2018^47^	Washing parameters
				Zambrano et al., 2021^60^	Fabric parameters
*Environmental Science and Pollution Research*	Environmental Science	Environmental Chemistry, Pollution	5	Carney Almroth et al., 2018^13^	Fabric parameters
				Hazlehurst et al., 2023^48^	Washing parameters/fabric parameters/test method
				Kärkkäinen and Sillanpää, 2021^55^	Efficiency of microfiber capture
				Rathinamoorthy and Raja Balasaraswathi, 2022^61^	Fabric parameters
				Rathinamoorthy and Raja Balasaraswathi, 2023^62^	Fabric parameters
*ACS Environmental Science and Technology*	Environmental Science	Chemistry, Environmental Chemistry	3	De Falco et al., 2020^38^	Fabric parameters
				Hernandez et al., 2017^49^	Washing parameters/fabric parameters
				Kelly et al., 2019^41^	Washing parameters
*PLoS One*	Multidisciplinary	Multidisciplinary	3	Browne et al., 2020^56^	Efficiency of microfiber capture
				Lant et al., 2020^42^	Washing parameters
				Vassilenko et al., 2021^44^	Fabric parameters
*The Journal of the Textile Institute*	Material Science	Polymers and Plastics, Materials Science	3	Özkan and Gündogdu, 2020^14^	Fabric parameters
				Raja Balasaraswathi and Rathinamoorthy, 2021^63^	Fabric parameters
				Rathinamoorthy and Raja Balasaraswathi, 2021^69^	Washing parameters
*Marine Pollution Bulletin*	Environmental Science	Oceanography, Pollution	2	Napper and Thompson, 2016^12^	Washing parameters/fabric parameters
				Zambrano et al., 2019 (^64^)	Fabric parameters
Scientific Reports	Multidisciplinary	Multidisciplinary	2	De Falco et al., 2019^50^	Washing parameters
				Volgare et al., 2021^51^	Washing parameters
Water, Air and Soil Pollution	Environmental Science	Environmental Chemistry, Pollution	2	Dalla Fontana et al., 2021^65^	Fabric parameters
				De Falco et al., 2021^57^	Efficiency of microfiber capture
*AATCC Journal of Research*	Material Science	Materials Chemistry, Polymers, and plastics	1	Frost et al., 2020^66^	Fabric parameters
*Dyes and Pigments*	Chemical Engineering	Process Chemistry and Technology, Chemical Engineering	1	Cotton et al., 2020^52^	Washing parameters
*Journal of Cleaner Production*	Environmental Science	Environmental Science	1	Cai et al., 2020^67^	Fabric parameters
*Science of the Total Environment*	Environmental Science	Environmental Chemistry, Pollution	1	Napper et al., 2020^58^	Efficiency of microfiber capture
*Sustainability*	Environmental Science	Environmental Science	1	Jönsson et al., 2018^68^	Test method
*Process Safety and Environmental Protection*	Multidisciplinary	Multidisciplinary	1	Jiang et al., 2022^53^	Washing parameters
*Polymers*	Multidisciplinary	Multidisciplinary	1	Choi et al., 2022^54^	Washing parameters/fabric parameters

The fact that researchers have differing aims of the research is important when discussing the methodology used, the information shared within the methodology, the format of result and the potential reasons for results found. As this is an emerging area of research it is natural that comparability may be compromised as “whenever a new form of contamination is discovered, it is inevitable that in the early stages of research a variety of methods will be applied”.^25^ This information is important for the discussion provided in the following.

**Figure 3:**
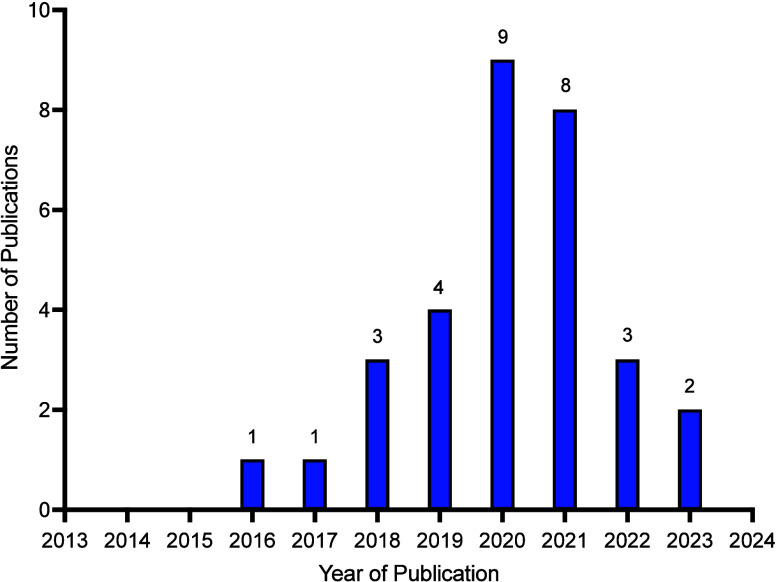
Graph displaying year of acceptance against the 32 screened papers identified during the systematic literature search.

Figure 3 highlights that the number of papers increased year on year until 2020 which reflects the growing importance of this field of research. The slight drop in numbers could be linked to the COVID-19 pandemic, during which review processes have taken longer and/or research diversifying into non fashion/textile contexts. The journals in which the systematically selected papers were published are shown in Table 2. A majority of these fall within the environmental science category, rather than fashion and/or textile focused journals.

The remainder of this article will analyze the different knit fabric parameters that have been studied within the systematically selected research. This is due to the emerging need for concise information for textile manufactures to create textiles that shed less microfibers while still being economically and aesthetically appealing.^1^

## Discussion

Studies show significant variation on techniques used to assess microfiber shedding rates from laundering of clothes and textiles, hence results have varied.^25^ As microfiber pollution from clothing and textiles is an emerging area of research, it is not detrimental that studies have chosen differing methods. Research investigating microfiber pollution of fabrics requires detailed understanding of textile processes as well as knowledge of analytical chemistry procedures, thus there are multifaceted complexities to this research and studies are being conducted from different viewpoints. To combat these complexities, comparisons between results will be shown when possible and areas for standardization within methodology will be highlighted as a guidance to ensure results of future studies can be compared with greater ease. Additionally, future research agendas that could lead to interventions of this pollution through sustainable fabric and clothing design are discussed.

### Effect of Fabric Parameters (Polymer, Yarn, Fiber) on Microfiber Shedding

#### Effect of Polymer on Microfiber Shedding

Various studies were related to assessing the emissions of microfibers from different knit fabrics. From these studies it was shown that fabric parameters such as the polymer of the fiber used impacts microfiber release.^14,38,58,64,66^ It is important to note here that most articles reviewed either refer to staple and/or filament fibers, or polyester and cotton rather than to any other polymers (e.g., polyamide, acrylic). A reason for this phenomenon could be that the focus was on clothing and textiles. To explain, within the textile and apparel industry polyester and cotton are dominating the market (Textile Exchange, 2021.^70^ This provides an opportunity for future research to conduct a SLR focusing on different polymers (e.g., natural, synthetic, semisynthetic).

Polyester is the most used fabric within the textile and fashion industry, which is reflected in the fact that from the systematically selected studies polyester was the most tested polymer, with 26 out of 32 studies using a polyester fabric sample as at least one of their test fabrics either in pure form or as a blend.^14,46,56,70^ A further explanation could be that polyester is known to have a high shedding rate and thus warrants further analysis to significantly reduce pollution.^27^ The second most dominant fiber used for the washing experiments was cotton which again reflects the manufacturing and clothing market.^70^ Zambrano et al.^60^ focused on 100% cotton knit fabrics with different finishes and found that fabrics treated with silicon softener and durable press released more fibers than untreated fabric.

Some studies pursued to distinguish microfiber shedding rates between different polymers or polymer blends.^14,38,54,58,64,66^ For example, Özkan and Gündoğdu^14^ noted that recycled polyester knit fabrics released almost 2.3 times more fibers than virgin polyester fabrics owed to the recycling process and the impact on the fiber’s tenacity and therefore its resistance to the washing processes ability to break off fiber fragments. Furthermore, the fibers released were also noted as being shorter on average due to a reduced tensile strength and increased hairiness of the recycled yarn. Although slightly out of scope for this SLR, future research could explore this further, as recycled yarns are often listed as “more sustainable” alternatives for the textile industry and consumers,^32^ yet this could be misleading if all aspects of its environmental impact are not fully understood.

When varying blends of recycled and virgin polyester knit fabrics were investigated it was found that a 70% blend of recycled polyester fabrics shed significantly less microfibers than a 40% blend of recycled polyester fabrics.^66^ These results contradicted Frost et al.’s hypothesis and Özkan and Gündoğdu’s findings that higher percentage blends of recycled fibers would shed greater numbers of microfibers due to lowered tensile strength.^14,66^ The contrast in results is likely because all yarns studied by Frost et al. had varying recycled polyester content as well as differing twists per meter which “were outside the scope of this study, yet they may have influenced the shedding propensity of the fabrics”.^66^ This highlights the need for systematic studies with singular parameter changes to assess individual influences on microfiber shedding.

Similarly, Zambrano et al. found that “fabrics made of cellulose-based fibers (cotton and rayon) release[d] more microfibers than polyester during laundering”,^64^ which was owed to yarn and fiber physio-chemical properties. Within the systematically selected studies, research that examined yarn and fiber physio-chemical properties theorized that fiber fragments release was a correlation to the yarns tendency to pill formation.^12,53,64^ Polyester was said to have a higher yarn breaking strength and abrasion resistance, which led to less fiber fragments being released compared to cellulose-based fibers.^64^ This was echoed by Choi et al.^54^ who found when keeping the knit structure consistent, and thus assessing the physical properties of fabrics that affect fiber release, polyester released the highest amount of fiber fragments during washing followed by acrylic and nylon. It was concluded that “fabrics with a higher yarn breaking strength, abrasion resistance, and flexural stiffness are expected to have a lower tendency to form fuzz or to release microfibers during the mechanical action of washing”.^54^

To further this, from a 50:50 blend polyester-cotton knit fabric 80% of the microfibers released were identified to be cotton due to the differences in tensile strength and fiber characteristics such as fiber length.^38^ Cotton has a lower tenacity compared to the synthetic alternatives, and thus it would be expected to see a greater number of cotton microfibers shed compared to polyester due to increased breakage and pill break-off.^64^ Yet within the systematically selected literature, it was shown that polyester-cotton blended fabrics released fewer microfibers than 100% acrylic and 100% polyester fabrics.^12^ These findings are contradictory to Zambrano et al. and De Falco et al.^38,64^ Notably, the materials examined within these studies were sourced through high-street stores and thus the differences in findings seen were suggested to be due to potential modifications of the synthetic materials surface.

In support of this, Dalla Fontana et al.^65^ found there was no correlation to tendency of yarn to pill and microfiber release. However, in contrast Zambrano et al.^64^ noted how microfiber release can be correlated to pill and fuzz formation and thus breaking strength and tensile strength. In relation to marketing and an “aesthetic perspective, there may be benefits to the release of pills from garments during washing for aesthetic purposes”.^12^ which should be considered. Contrasting results between Zambrano et al.^64^ and Dalla Fontana et al.^65^ could be due to multiple parameters such as edging techniques, yarn type, linear mass density, and pilling tendency changing between the two fabric samples within the Dalla Fontana et al.^46^ study. With multiple parameters being inconsistent, this could have masked potential influences of pilling tendency. This highlights that future studies should aim to keep as many of the fine scale parameters consistent as possible.^25^

Alongside quantity, few studies investigated the length of shed microfibers and how the polymer of the knit fabrics impacted length of microfibers shed.^14,25,44,54,66^ From an environmental contamination perspective, the length of fibers shed is important to understand as the smallest of fibers are more likely to pass through filter devices fitted within or on wastewater pipes of washing machines, they are also more likely to be ingested by marine and terrestrial organisms.^17,29^ Thus, a better understanding of the average lengths alongside upper and lower limits of shed microfibers can help create effective filtering devices.

Within the systematically selected studies, there was a lack of clear consensus of how the polymer of the knit fabric impacts the length of microfibers shed. For example, Vassilenko et al.^44^ found there to be no significant difference among length of fibers shed from cotton, wool, virgin polyester, recycled polyester and virgin nylon. However, it should be noted that recycled nylon released significantly longer fibers compared to the other samples. This was also found by Frost et al.^66^ whereby the mean length of microfibers shed from fabrics made of 70% recycled polyester were significantly longer than virgin polyester.

This contrasts with other work such as De Falco et al.^38^ and Choi et al.^54^ whereby cotton knit fabrics shed longer fibers than polyester, while acrylic knit fabric shed longer fibers than nylon. Both studies owed the differences in lengths between the polymers due to the chemical composition and breaking strength. Choi et al. explains that “this result is attributed to the resistance to washing friction being different for each fabric component due to the different physical properties of each fabric”.^54^ Additionally, Özkan and Gündoğdu noted that microfibers shed from recycled polyester were significantly shorter than virgin polyester which was owed to the “reduced strength of [recycled polyester] by the recycling process against the thermomechanical effects in the washing process”.^14^ Physical properties of yarn and fabrics can be very complex and influenced by hydrophilicity, wettability, and creation technique which might explain contrasting results seen within studies.

There is not a clear consensus within the literature on the effect of polymers on microfiber shedding (Table 3). A clear conclusion on which polymers release fewest microfibers cannot be drawn due to differing sources of fabrics or the myriad of parameters that were altered between assessed fabrics, with polymers of yarn being one of them. This highlights the need for methodical and systematic studies using standardized methodology to test and measure microfiber pollution.

**Table 3:** Summary of Current Research Findings from the Systematically Selected Articles

Category		Findings	Publication (Reference)	Considerations, Reasonings for Contrasting Results, and Future Research Agendas
Fiber	Polymer	Cellulose textiles released more microfibers than synthetic textiles	Zambrano et al., 2019^64^	It is suggested that the difference in results from Napper et al.^58^ and Zambrano et al.^64^ to be due to possible modification of fabric due to purchasing of fabrics on the high street therefore future studies should aim to create textiles in house to allow full history of textiles to be known.
		Cellulose textiles released fewer microfibers than synthetic textiles	Napper et al., 2020^58^	
		Recycled polyester shed more microfibers than virgin polyester	Özkan and Gündoğdu, 2020^14^	Future research agendas should focus on emerging textile polymers (i.e., orange, pineapple fibers etc.) and assess full impact of different polymers and blends that are used in highstreet clothing (i.e., acrylic, polyamide).
		Yarns with greater % blends of recycled polyester mixed with virgin polyester content release less microfibers than blends with lower percentages	Frost et al., 2020^66^	Conflicting results of cellulose vs synthetic and recycled polyester should be further studied by keeping as many other fabric parameters such as twist of yarn the same, with only polymer of yarn changing.
		Microfiber release can be correlated to pill and fuzz formation (and thus breaking strength and tensile strength).	Zambrano et al., 2019^64^	Contrasting results between Zambrano et al.^64^ and Dalla Fontana et al.^65^ could be due to multiple parameters changing within sampled fabrics causing issues when identifying influence to microfiber shedding.
		Pilling and fuzz formation does not correlate to microfiber release during washing.	Dalla Fontana et al., 2021^65^	Future research agendas should focus on systematically altering singular fabric parameters as possible.

This study also indicates that there is a gap within the systematically selected studies in regard to standardized methods and comparable results which is due to the recent advancements of testing standards.^8,71^ This has impacted advancements in eco-design measures to be suggested as a lack of concise or comparable findings, which has been a “major barrier to both regulatory and voluntary action”.^72^ An extended producer responsibility policy or VAT reduction in favor of reducing microfiber pollution has been suggested by Eunomia “dependent, of course, upon an appropriate measurement method”.^30^ This both highlights the strive toward eco-design of clothing and the need for a standardized measurement methodology.^1,73^

In the future, with trends in the fashion industry moving away from either resource intensive fibers (e.g., cotton) or those reliant on petroleum (e.g., polyester), we may see a shift in polymers available, which should also be reflected in research scopes. For example, the shedding rates and potential pollution sources from fabric made of recycled fibers, as well as man-made natural fibers (e.g., orange or pineapple fiber) could be explored.

Within this SLR, comparability between results is often hindered by published information on the fabric samples used. Napper and Thompson^25^ suggest that future research should record and publish parameters including: fiber type (e.g., cross-section shape, cross-section thickness, length, composition); yarn type (e.g., staple or filaments, number of filaments, twists per unit length); polymer (e.g., natural, synthetic, semisynthetic); fabric type (e.g., density, thickness, mass per unit area); condition (e.g., new, worn, aged); and description of textile material (e.g., garment or swatch, size, cutting mechanism, seaming procedure and total weight). Publishing this information, whether test methods are standardized or not will advance comparability between results which is a necessary step to move toward greater understanding of the potential voluntary and involuntary source-derived design interventions of microfiber pollution in textiles and apparel.

#### Effect of Yarn Physical Classifications on Microfiber Shedding

**Figure 4:**
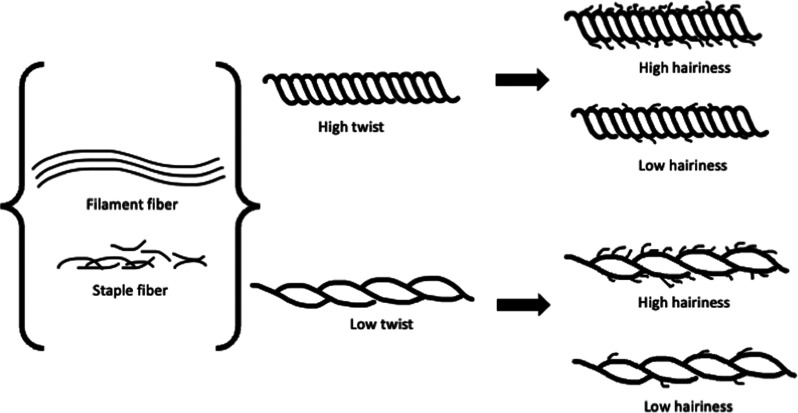
Diagram of stable and filament fibers, high twist and low twist yarn, and high and low hairiness variations. (Authors own representation).

The physical classification of yarns, aside from the polymer used (e.g., natural, synthetic, semisynthetic), includes characteristics such as length of fiber that make up the yarn (filament or staple), the twist of the fibers that hold the yarn together and hairiness of the yarn (shown in Figure 4). Often, all three of these characteristics are interconnected and each characteristic is often chosen by textile and clothing manufactures as the fiber length, twist and hairiness can affect the appearance and feel of an item.^14,29^

Most natural fibers, such as cotton and wool, are staple fibers.^74,75^ Staple fibers are defined as fibers of varying lengths which are spun and twisted together to make a continuous yarn used for knitting. Due to the short fiber length, fabrics made of staple fibers often have protrusion of fibers on the surface of the fabric which is known as hairiness, making a fluffier appearance and softer feel which is commonly used for winter clothing.^14,74,75^ Hairiness can also be impacted by how much the fibers are twisted together to create the fabric yarn.^75^

Filament fibers are of a continuous length and made from chemical fiber manufacturing processes to form synthetic fibers such as polyester and acrylic or from silkworms to form silk.^74−76^ Usually, a couple of filament fibers are twisted together to create yarn, which creates a smoother surface compared to fabric made of staple fibers.^75^ For aesthetic purposes, filament fibers can be cut to a desired length and twisted together to create yarn with a desired hairiness.^75^

Within the systematically selected studies, fabrics created from shorter staple fiber lengths that have been spun or twisted into yarn have been noted to relate to greater microfiber shedding during washing of knit fabrics compared to longer stable fibers or filament fiber yarns.^13,45,49^ This has been owed to “shorter staple fibers could more easily slip away from the yarn during the wash, leading to a higher microfiber release”.^47^ Additionally, the length of fibers (whether that be change in staple fiber length or change from filament fiber or staple fibers) impacts the hairiness of the fabric created which has been shown to have a positive correlation with higher rates of microfiber shedding.^64^ With higher hairiness increasing the number of fibers protruding from the surface of the fabric, this alludes to greater numbers of potential fibers being subjected to external shear forces during laundering that lead to fiber fragmenting and being released as microfiber pollution.^14,64^ Research conducted on filament and staple fibers indicated that alongside quantity of pollution, the length of microfibers released was affected by the length of the fibers within the yarn; for instance, it was shown that microfibers released from filament fiber fabrics were longer than the staple fiber samples.^14^

Switching to continuous filament fibers compared to staple fibers could “indicate possible changes in textile design for apparel industries, which could contribute to the reduction of microplastic release”.^50^

In contrast to previous research De Falco et al.^47^ and De Falco et al.^38^ found that polyester filament knitted fabric shed more microfibers than polyester staple knitted fabric. However, due to the experimental design and the fabrics examined having multiple parameters at change, the differences in microfibers shed could not be alluded to the influence of the fiber lengths as the polyester filament yarn. The filament yarn had lower twist and greater hairiness in comparison to the polyester staple yarns showing that higher hairiness would have favored microfiber release.^47^ For De Falco et al.^38^ the fabric made of continuous filament yarn which shed more microfibers in comparison had no twist and low hairiness while the staple yarn had moderate twist to the yarn and high hairiness. Due to the higher hairiness fabric shedding less microfibers (and thus in contrast to previous work) the microfiber release was attributed to the addition of twists to the yarn which reduced the amount of pollution release during washing. A future research scope should address which textile parameters has the greatest influence on microfiber shedding, such as staple or filament fiber, twist, and hairiness of the yarn.

Similarly, Hazlehurst et al. noted that “several staple fiber fabrics did not meet the hypothesis [that fabrics constructed of staple yarns would shed more than fabrics constructed of filament fibers] having very low release rates compared to some filament fiber fabrics”.^48^ This was owed to differences in fabric structure and finishing technique, and thus does not discredit previous work that fabrics created of staple fibers shed more than filament fibers but highlights the complexity of each textile parameter influencing microfiber shedding.

This furthermore conveys the demand for studies to systematically alter individual parameters to allow for results to be unambiguous which will allow clear directives to be followed by textile and apparel manufactures who aim to reduce microfiber shedding from fabrics during laundering.

Additionally, research has noted that stress built into the yarn through spinning technique to create the desired twist could be an indication factor of level of microfiber shedding during laundering of finished garments.^49^ This highlights that the mitigation of microfiber shedding can be addressed across the industry within all stages.

From the systematically selected studies, there is a gap in research in how these intervention points to reduce microfiber shedding will be communicated, implemented, and controlled within the textile and apparel industry. Incorporating the environmental pollution released from garments over their lifetime, and other circular economy principles, will have certain challenges such as business model innovation, regulatory pressures, financial pressures, and consumer related issues.^77^ Future research should provide insight into how best to distribute the information surrounding the mitigation strategies of the release of microfiber pollution to fabric and clothing manufacturers alongside consumers and other stakeholders to initiate effective change.

In summary, the systematically selected studies are somewhat conclusive in that eco-design plays a key role and measures need to be taking related to physical yarn classifications, including, but not limited to length of fiber, yarn twist, and yarn hairiness. As previously outlined, each of these yarn classifications can have an impact on reducing the amount of fiber fragments released, especially during the laundering process (see Table 4). Yet, findings are only somewhat conclusive, which implies that there are also various limitations within each of the studies analyzed that make it challenging to provide an actual comparison (e.g., multiple versus single parameter changes). Thus, further research is needed to systematically assess yarn parameters, individually and in combination, to assess their proportional relationship to microfiber shedding.

**Table 4:** Summary of Current Research Findings from the Systematically Selected Articles

Category		Findings	Publication (Reference)	Considerations and Future Research Agendas
Polymer	Length of fiber (staple vs filament)	Shorter fibers shed more as they protrude more out of the fabric structure and therefore are easier to be fragmented or released	Cesa et al., 2020^45^	Future research agendas should focus on how best to distribute information on eco-design for microfiber shedding to clothing designers, manufactures and consumers.
			De Falco et al., 2018^47^	
	Yarn twist	Highly twisted yarns release less microfibers as less fibers protrude from fabric structure and are held into structure in tighter twists than loose/no twist yarn	De Falco et al., 2019^50^	Education on the look or “fluffiness” of a knitted structure and its correlation to microfiber pollution could be a good starting point for consumer education and conscious buying.
			Carney Almroth et al., 2018^13^	
	Yarn hairiness	Low hairiness yarn releases less microfibers as less fibers protrude from surface of fabric structure	Zambrano et al., 2019^64^	
			Carney Almroth et al., 2018^13^	

This could have implications for practitioners (e.g., manufactures, designers) in the future. To explain, “textiles” (here used loosely) are often chosen for their aesthetic properties (e.g., hairiness for winter jumpers) rather than on the basis of their microfiber shedding rate. Similarly, filament and/or staple fibers are selected for their specific properties and client requirements, which could make it challenging to enforce change to use “textiles” that are shedding less microfibers and thus, are part of an eco-design process.

#### Effect of Fabric Construction on Microfiber Shedding

Another source directed intervention during the design stage of textile and clothing manufacturing is the compactness of the fabric. However, possibly due to differences in methodologies there are conflicting results within the studies and “influence of knit structure [on microfiber shedding] is not entirely clear”.^78^

Increasing the stitch density and therefore increasing the tightness factor of the fabric has been shown to reduce the microfibers shed during laundering due to the tighter structure lowering the probability of fibers slipping out of the structure.^63^ This is also shown by looser structured knits shedding more fibers when compared to tighter knit structures.^55^ When comparing a “fluffy” knit jumper to a tighter knit t-shirt, Kärkkäinen and Sillanpää found “looser fibers [are] susceptible to being broken off from textile surface, for example due to mechanical stress from washing”.^55^ With increased education and public awareness consumers could be capable to make more informed or eco-conscious buying decisions if the link between hairiness and microfiber shedding was clearly defined due to the tactile nature of “fluffy” “fuzzy” knitwear when compared to less hairy knitwear.^73,79^

Contrary to Kärkkäinen and Sillanpää,^55^ it has been shown that increasing density of knit fabrics increases microfiber pollution released during washing.^13,59^ Carney Almroth et al. stated, “more tightly knitted fabric, as indicated by the knitting gauge results in more fibers on the same area of fabric resulting in a greater fiber loss”.^13^ However, De Falco et al. noted “the microfibers released could not be related to the number of fibers present per unit area, since [double jersey knit polyester fabric swatch], that has the greatest weight, is also the fabric that released less microfibers”^47^ showing that further clarity through systematic research is needed. This is an area for future research which if understood could be used to tailor policies and sustainable incentives.

From the studies selected for this SLR it is evident that fabric characteristics that influence microfiber shedding have a complex relationship. Cesa et al. outlines that on the “one side textile parameters linked to mass of fibers (i.e., fabric weight per unit area, fabric thickness, linear density or yarn count) make more material available, on the other side, those responsible for fibers cohesion (i.e. fabric density, fabric interlacing, yarn twist, fibers size and regularity) hold it, avoiding propagation”.^45^ It is noted how often studies have tried to characterize the relationships between these parameters but are “neither exhausted nor isolated, they suggest clues in this type of pollution”.^45^ This SLR highlights that majority of research does not operate “controlled” manufacturing and thus future research should focus on utilizing under instruction or in-house construction of fabrics to allow for individual parameters such as textile compactness to be assessed. Furthermore, it is imperative that each research study clearly outlines the fiber, yarn, and fabric parameters alongside how swatches are created to allow for greater comparability between studies. This information is important to allow textile products to be designed to release as little fibers as possible.

A limitation of manipulating fabric construction will be how these can be translated and marketed for consumer use.^77^ For instance, changing the tightness of the knit can impact how the fabric feels, sits, and the cost of the materials. However, future research should continue to assess how better design can mitigate microfiber pollution as “any further measures down stream of production (be they in machine type, wash cycle, chemicals used or filtration and collection devices) will exert their effect on top of the reductions achieved by better design”.^25^ Without clear and concise actions that can be taken from each manufacturing stage such as yarn selection (polymer type, yarn twist, yarn hairiness and fiber length) and/or fabric construction (knit structure, tailoring technique), microfiber pollution will continue to be released into our environment at alarming rates.

Few papers examined how the tailoring process of creating garments influences the amount of pollution released during laundering.^63,65,67,68^ Cai et al. noted that “scissor-cut textiles demonstrated three to 31 times higher number of extracted MPFs than laser-cut textiles”.^67^ Dalla Fontana et al.^65^ and Jönsson et al.^68^ agreed that microfibers are lost from cut fabric during washing, and thus to reduce microfiber pollution edges should be double folded over or heat cut/sealed. This shows that textile and garment manufactures can implement tailoring techniques that could release less microfiber pollution than other techniques used.

Recently, modification and finishing processes have been suggested to be used to mitigate microfiber release.^60−62^ Capitalizing on already used processes in the textile industry (softeners, durable press) or inventive coatings (enzyme hydrolysis), their interrelationship with microfiber pollution could help aid microfiber mitigation. Enzyme hydrolysis on polyester knitted fabric was shown to significantly reduce microfiber shedding over 20 washing cycles.^61^ On the other hand, textiles treated with a silicon softener and durable press were shown to generate more microfibers during laundering than untreated fabrics.^60^

Overall, the systematically selected studies provide comprehensive investigations into microfiber release dependencies on yarn characteristics and properties, as well as fabric structure. Raja Balasaraswathi and Rathinamoorthy^63^ concluded that fabric parameters such as thickness, tightness, and stitch density were of greater importance to influencing microfiber release compared to physical fabric properties (tensile strength, pilling resistance). However, there is a research gap within the systematically selected studies on the hierarchy of influence of microfiber release and the parameters of textile that have the greatest influence during laundering should be high priority for future research alongside understanding contradicting conclusions as discussed within this literature review. This review suggests that the release of microfibers is not driven by individual factors but work in combination, with complex relationships that need further investigation to fully understand.

Thus far, the review has highlighted that microfiber pollution is a complex issue. There are various aspects including but not limited to yarn, fiber, and polymer used that could act as mitigation strategies and thus reduce the amount of fiber shed. Yet, it is also apparent that there are inconclusive results overall, as different studies often have different outcomes and as such general statements can currently not be made. What is apparent however is that more research is needed to verify and solidify outcomes especially focusing on different yarn parameters.

With microfiber pollution still being a relatively recent topic, with its impact on the natural environment remaining uncertain, especially when it comes to indigestion, more research needs to be done that focuses on a common stakeholder approach. As alluded to in the SLR, even if it is evidenced that some structures may shed less microfibers, consumers may not necessarily buy into these products, due to aesthetics and/or feel. Yet, if communication strategies would center around the benefits of certain knit structures, due to reduced microfiber shedding, stakeholders, and more specifically consumers, may show more of a buy-in. This, however, needs to be further verified with primary data collection.

### Implications

This SLR highlights that yarn, fiber and textile construction are important factors to impact the quantities of microfibers shed during washing (Table 5). As recalled by Liu et al. “multiple stakeholders in the fashion supply chain contribute to solving the problem of microfiber pollution” and it is suggested that “improvements in the properties of fiber, yarn, and fabric in the design and production stage are the most effective methods to limit microfiber emissions”.^80^

**Table 5:** Summary of the Eco-design Methods to Reduce Microfiber Shedding from Textiles from the Systematically Selected Research Articles

Category		Findings	Publication (Reference)	Considerations and Future Research Agendas
Fiber	Polymer	Cellulose textiles released more microfibers than synthetic textiles	Zambrano et al., 2019^64^	Napper et al.^58^ suggested findings to be due to possible modification of fabric therefore future studies should create textiles in house to allow full history of textiles to be known.
		Cellulose textiles released fewer microfibers than synthetic textiles	Napper et al., 2020^58^	
		Recycled polyester shed more microfibers than virgin polyester	Özkan and Gündoğdu, 2020^14^	Future research agendas should focus on emerging textile polymers and assess full impact of different polymers and blends that are used in highstreet clothing.
		Yarns with greater % blends of recycled polyester mixed with virgin polyester content release less microfibers than blends with lower percentages	Frost et al., 2020^66^	Conflicting results of cellulose vs synthetic and recycled polyester should be further studied.
	Length of fiber (staple vs filament)	Shorter fibers shed more as they are easier to be fragmented or released from textile structure	Cesa et al., 2020^45^	Future research agendas should focus on how best to distribute information on eco-design for microfiber shedding to clothing designers, manufactures and consumers.
			De Falco et al., 2018^47^	
	Yarn twist	Highly twisted yarns release less microfibers than loose/no twist yarn	De Falco et al., 2019^50^	
			Carney Almroth et al., 2018^13^)	
	Yarn hairiness	Low hairiness yarn releases less microfibers	Zambrano et al., 2019^64^	Conflicting results of impact of knit construction should be further studied.
			Carney Almroth et al., 2018^13^	
Fabric construction	Gauge of knit	Looser knit structure shed more microfibers than tighter structures	Kärkkäinen and Sillanpää, 2021^55^	Future studies should isolate and systematically assess relationships between microfiber pollution and construction parameters.
			Raja Balasaswathi et al., 2021^63^	
		Looser structures shed less microfibers than tight knit structures	Belzagui et al., 2019^59^	
			Carney Almroth et al., 2018^13^	

However, complexities are prevalent when assessing the routes of intervention for reducing microfiber shedding through the design and production stage of textiles and apparel. This has been coupled by lack of detailed fabric parameters or wash settings outlined within studies and absence of standardized methodology for testing (Table 6), which has hindered comparability and led to high uncertainty of the proportional influences of this pollution source.^1^

**Table 6:** Summary of the Suggest Method Standardization and Advancement and the Benefits of This to Microfiber Pollution Assessments

Category		Method Standardization/Advancement	Benefits of Method Standardization/Advancement	Considerations and Future Research Agendas
Fabric	Source of fabric or garment	Future research should utilize use in-house construction or instructed construction of fabrics	This will allow greater control of subtle changes to fabric parameters that influence microfiber pollution and greater knowledge of treatment of fabrics	Must be able to scale findings to be relevant for mass production of clothing
				Clear detailed information on fabric parameters should be published for comparability between studies
	Polymer and fabric selection	No standardization recommendations for polymer selection as based on research aim	Brightly colored fabrics are easier to visually identify, can be distinguished from contamination which reduces miscounting	Clear detailed information on fabric parameters should be published for comparability between studies
		Use bright colored fabrics	This will allow greater control of subtle changes to fabric parameters that influence microfiber pollution and greater knowledge of treatment of fabrics	Future studies should assess emerging fabric compositions alongside existing ones
		Future research should utilize use in-house construction or instructed construction of fabrics		
	Size of textile used	No broad standardization recommendations as based on research aim and wash equipment used		Future studies need to consider how findings will be scaled to real consumers washing so that policies can be clearly define
				Clear detailed information on fabric parameters, cutting technique and seam process should be published for comparability between studies
				Future studies should assess how surface area, weight, density, and overall size of fabric interact with microfiber release rates

From the systematically selected studies, it is apparent more research is needed to draw robust conclusions on the relationship between individual textile parameters and microfiber pollution. We conclude that future research should ensure that information such as yarn type, twist, filament or staple, fabric structure, and type are recorded and detailed within the published research to allow for greater comparability and conclusions to be drawn between studies.

The confirmation of how stakeholders can shift design and production of textiles and apparel to reduce microfiber pollution throughout the garment’s lifetime is a fundamental parameter to moving toward controlling pollution from the source. While there are currently no regulations on industry standards to monitor or reduce microfiber pollution, with actionable areas of interest such as yarn type and fabric structure, governments can intervene and hold the textile industry accountable through voluntary and involuntary means to ensure the proliferation of microfiber pollution is controlled and monitored from the source.

## References

[ref1] EEA (European Environment Agency). Microplastics from textiles: towards a circular economy for textiles in Europe. https://www.eea.europa.eu/publications/microplastics-from-textiles-towards-a (accessed 2023–04–06).

[ref2] Browne M.; Crump P.; Niven S.; Teuten E.; Tonkin A.; Galloway T.; Thompson R. Accumulation of Microplastic on Shorelines Worldwide: Sources and Sinks. Environ. Sci. Technol. 2011, 45 (21), 9175–9179. 10.1021/es201811s. 21894925

[ref3] Thompson R.; Olsen Y.; Mitchell R.; Davis A.; Rowland S.; John A.; et al. Lost at Sea: Where Is All the Plastic?. Science 2004, 304 (5672), 838–838. 10.1126/science.1094559. 15131299

[ref4] Athey S. N.; Erdle L. M. Are We Underestimating Anthropogenic Microfiber Pollution? A Critical Review of Occurrence, Methods, and Reporting. Environ. Toxicol. Chem. 2022, 41, 822 10.1002/etc.5173. 34289522

[ref5] Henry B.; Laitala K.; Klepp I. Microfibers from apparel and home textiles: Prospects for including microplastics in environmental sustainability assessment. Science Of the Total Environment 2019, 652, 483–494. 10.1016/j.scitotenv.2018.10.166. 30368178

[ref6] Sorensen R. M.; Jovanovic B. From nanoplastic to microplastic: A bibliometric analysis on the presence of plastic particles in the environment. Mar. Pollut. Bull. 2021, 163, 111926 10.1016/j.marpolbul.2020.111926. 33348287

[ref7] Yan S.; Jones C.; Henninger C. E.; McCormick H. Textile Industry Insights Towards Impact of Regenerated Cellulosic and Synthetic Fibres on Microfiber Pollution. Journal of Fashion Marketing and Management: An International Journal 2020, 24 (3), 437–454. 10.1108/JFMM-08-2019-0181.

[ref8] AATCC (American Association of Textile Chemists and Colorists). 2022 Manual of International Test Methods and Procedures, 97th ed.; 2022.

[ref9] Hari P. K. Types and properties of fibers and yarns used in weaving. Woven Textiles 2012, 3–34. 10.1533/9780857095589.1.3.

[ref10] Jamieson A. J.; Brooks L. S. R.; Reid W. D. K.; Piertney S. B.; Narayanaswamy B. E.; Linley T. D. Microplastics and synthetic particles ingested by deep-sea amphipods in six of the deepest marine ecosystems on Earth. Royal Society Open Science 2019, 6 (2), 180667 10.1098/rsos.180667. 30891254 PMC6408374

[ref11] Napper I. E.; Davies B. F. R.; Clifford H.; Elvin S.; Koldewey H. J.; Mayewski P. A.; Miner K. R.; Potocki M.; Elmore A. C.; Gajurel A. P.; Thompson R. C. Reaching New Heights in Plastic Pollution—Preliminary Findings of Microplastics on Mount Everest. One Earth 2020, 3 (5), 621–630. 10.1016/j.oneear.2020.10.020.

[ref12] Napper I.; Thompson R. Release of synthetic microplastic plastic fibers from domestic washing machines: Effects of fabric type and washing conditions. Mar. Pollut. Bull. 2016, 112 (1–2), 39–45. 10.1016/j.marpolbul.2016.09.025. 27686821

[ref13] Carney Almroth B. M.; Åström L.; Roslund S.; Petersson H.; Johansson M.; Persson N. K. Quantifying shedding of synthetic fibers from textiles; a source of microplastics released into the environment. Environmental Science and Pollution Research 2018, 25 (2), 1191–1199. 10.1007/s11356-017-0528-7. 29081044 PMC5766707

[ref14] Özkan İ.; Gündoğdu S. Investigation on the microfiber release under controlled washings from the knitted fabrics produced by recycled and virgin polyester yarns. Journal of The Textile Institute 2021, 112 (2), 264–272. 10.1080/00405000.2020.1741760.

[ref15] Boucher J.; Friot D. Primary Microplastics in the Oceans: A Global Evaluation of Sources; IUCN: Gland, Switzerland, 2017. 10.2305/IUCN.CH.2017.01.en.

[ref16] Athey S. N.; Carney Almroth B.; Granek E. F.; Hurst P.; Tissot A. G.; Weis J. S. Unravelling Physical and Chemical Effects of Textile Microfibers. Water 2022, 14 (23), 3797 10.3390/w14233797.

[ref17] Kwak J. I.; Liu H.; Wang D.; Lee Y. H.; Lee J. S.; An Y. J. Critical review of environmental impacts of microfibers in different environmental matrices. Comparative Biochemistry and Physiology Part C: Toxicology & Pharmacology 2022, 251, 109196 10.1016/j.cbpc.2021.109196. 34601087

[ref18] Rebelein A.; Int-Veen I.; Kammann U.; Scharsack J. P. Microplastic fibers - Underestimated threat to aquatic organisms?. Sci. Total Environ. 2021, 777, 146045 10.1016/j.scitotenv.2021.146045. 33684771

[ref19] Sharma M. D.; Krupadam R. J. Adsorption-desorption dynamics of synthetic and naturally weathered microfibers with toxic heavy metals and their ecological risk in an estuarine ecosystem. Environmental Research 2022, 207, 112198 10.1016/j.envres.2021.112198. 34656635

[ref20] Barrows A. P. W.; Cathey S. E.; Petersen C. W. Marine environment microfiber contamination: Global patterns and the diversity of microparticle origins. Environ. Pollut. 2018, 237, 275–284. 10.1016/j.envpol.2018.02.062. 29494921

[ref21] Gago J.; Carretero O.; Filgueiras A.; Viñas L. Synthetic microfibers in the marine environment: A review on their occurrence in seawater and sediments. Mar. Pollut. Bull. 2018, 127, 365–376. 10.1016/j.marpolbul.2017.11.070. 29475673

[ref22] Jensen L. H.; Motti C. A.; Garm A. L.; Tonin H.; Kroon F. J. Sources, distribution, and fate of microfibers on the Great Barrier Reef, Australia. Sci. Rep. 2019, 9, 9 10.1038/s41598-019-45340-7. 31227771 PMC6588688

[ref23] Leslie H. A.; Van Velzen M. J. M.; Brandsma S. H.; Vethaak A. D.; Garcia-Vallejo J. J.; Lamoree M. H. Discovery and quantification of plastic particle pollution in human blood. Environ. Int. 2022, 163, 107199 10.1016/j.envint.2022.107199. 35367073

[ref24] EC (European Commission). EU Strategy for Sustainable and Circular Textiles, 2022. https://environment.ec.europa.eu/publications/textiles-strategy_en. (accessed on 2023–04–09).

[ref25] Napper I. E.; Thompson R. C. Microfiber Shedding from Textiles during Laundering: Differing quantification methods but common findings. In Polluting Textiles: The Problem with Microfibers; Weis J., Ed.; Routledge, 2022; pp 135–152. 10.4324/9781003165385-8.

[ref26] Le L. T.; Nguyen K. Q. N.; Nguyen P. T.; Duong H. C.; Bui X. T.; Hoang N. B.; Nghiem L. D. Microfibers in laundry wastewater: Problem and solution. Sci. Total Environ. 2022, 852 (8), 158412 10.1016/j.scitotenv.2022.158412. 36055511

[ref27] McIlwraith H. K.; Lin J.; Erdle L. M.; Mallos N.; Diamond M. L.; Rochman C. M. Capturing microfibers - marketed technologies reduce microfiber emissions from washing machines. Mar. Pollut. Bull. 2019, 139, 40–45. 10.1016/j.marpolbul.2018.12.012. 30686443

[ref28] Erdle L. M.; Nouri Parto D.; Sweetnam D.; Rochman C. M. Washing Machine Filters Reduce Microfiber Emissions: Evidence From a Community-Scale Pilot in Parry Sound, Ontario. Frontiers in Marine Science 2021, 8, 777865 10.3389/fmars.2021.777865.

[ref29] De Falco F.; Cocca M. Innovative Approaches to Mitigate Microfiber Pollution. In Polluting Textiles: The Problem with Microfibers; Weis J., Ed.; Routledge, 2022; pp 245–264.

[ref30] Eunomia. Driving a CE for Textiles through EPR: Final Report, 2022; https://www.eunomia.co.uk/reports-tools/driving-a-circular-economy-for-textiles-through-epr/. (accessed 2023–06–09).

[ref31] Ellen MacArthur Foundation. The Nature Imperative: How the circular economy tackles biodiversity loss - Fashion. https://ellenmacarthurfoundation.org/biodiversity-report (accessed 2023–04–22).

[ref32] UNEP (United Nations Environment Programme) & UNFCCC (United Nations Framework Convention on Climate Change). The Sustainable Fashion Communication Playbook. https://www.unep.org/interactives/sustainable-fashion-communication-playbook/ (accessed 2023–06–10).

[ref33] Page M. J.; McKenzie J. E.; Bossuyt P. M.; Boutron I.; Hoffmann T. C.; Mulrow C. D.; Shamseer L.; Tetzlaff J. M.; Akl E. A.; Brennan S. E.; Chou R.; Glanville J.; Grimshaw J. M.; Hróbjartsson A.; Lalu M. M.; Li T.; Loder E. W.; Mayo-Wilson E.; McDonald S.; Moher D. The PRISMA 2020 statement: an updated guideline for reporting systematic reviews. BMJ 2021, n71 10.1136/bmj.n71. 33782057 PMC8005924

[ref34] Dissanayake K.; Pal R. Sustainability dichotomies of used clothes supply chains: a critical review of key concerns and strategic resources. International Journal of Logistics Management 2023, 34, 75–97. 10.1108/IJLM-10-2022-0410.

[ref35] Jagaba A. H.; Kutty S. R. M.; Noor A.; Birniwa A. H.; Affam A. C.; Lawal I. M.; Kankia M. U.; Kilaco A. U. A systematic literature review of biocarriers: Central elements for biofilm formation, organic and nutrients removal in sequencing batch biofilm reactor. Journal of Water Process Engineering 2021, 42, 102178 10.1016/j.jwpe.2021.102178.

[ref36] Rathinamoorthy R.; Raja Balasaraswathi S. A review of the current status of microfiber pollution research in textiles. International Journal of Clothing Science and Technology 2021, 33 (3), 364–387. 10.1108/IJCST-04-2020-0051.

[ref37] Cui H.; Xu C. Study on the Relationship between Textile Microplastics Shedding and Fabric Structure. Polymers 2022, 14, 5309 10.3390/polym14235309. 36501706 PMC9740661

[ref38] De Falco F.; Cocca M.; Avella M.; Thompson R. C. Microfiber Release to Water, Via Laundering, and to Air, via Everyday Use: A Comparison between Polyester Clothing with Differing Textile Parameters. Environ. Sci. Technol. 2020, 54 (6), 3288–3296. 10.1021/acs.est.9b06892. 32101431

[ref39] Galvão A.; Aleixo M.; De Pablo H.; Lopes C.; Raimundo J. Microplastics in wastewater: microfiber emissions from common household laundry. Environmental Science and Pollution Research 2020, 27 (21), 26643–26649. 10.1007/s11356-020-08765-6. 32378098

[ref40] Haap J.; Classen E.; Beringer J.; Mecheels S.; Gutmann J. Microplastic Fibers Released by Textile Laundry: A New Analytical Approach for the Determination of Fibers in Effluents. Water 2019, 11 (10), 2088 10.3390/w11102088.

[ref41] Kelly M. R.; Lant N. J.; Kurr M.; Burgess J. G. Importance of Water-Volume on the Release of Microplastic Fibers from Laundry. Environ. Sci. Technol. 2019, 53 (20), 11735–11744. 10.1021/acs.est.9b03022. 31460752

[ref42] Lant N. J.; Hayward A. S.; Peththawadu M. M. D.; Sheridan K. J.; Dean J. R. Microfiber release from real soiled consumer laundry and the impact of fabric care products and washing conditions. PLoS One 2020, 15 (6), e0233332 10.1371/journal.pone.0233332. 32502152 PMC7274375

[ref43] Rathinamoorthy R.; Raja Balasaraswathi S. Domestic Laundry and Microfiber Shedding of Synthetic Textiles. Microplastic Pollution 2021, 127–155. 10.1007/978-981-16-0297-9_5.

[ref44] Vassilenko E.; Watkins M.; Chastain S.; Mertens J.; Posacka A. M.; Patankar S.; Ross P. S. Domestic laundry and microfiber pollution: Exploring fiber shedding from consumer apparel textiles. PLoS One 2021, 16 (7), e0250346 10.1371/journal.pone.0250346. 34242234 PMC8270180

[ref45] Cesa F. S.; Turra A.; Checon H. H.; Leonardi B.; Baruque-Ramos J. Laundering and textile parameters influence fibers release in household washings. Environ. Pollut. 2020, 257, 113553 10.1016/j.envpol.2019.113553. 31761586

[ref46] Dalla Fontana G.; Mossotti R.; Montarsolo A. Assessment of microplastics release from polyester fabrics: The impact of different washing conditions. Environ. Pollut. 2020, 264, 113960 10.1016/j.envpol.2020.113960. 32375087

[ref47] De Falco F.; Gullo M.; Gentile G.; Di Pace E.; Cocca M.; Gelabert L.; et al. Evaluation of microplastic release caused by textile washing processes of synthetic fabrics. Environ. Pollut. 2018, 236, 916–925. 10.1016/j.envpol.2017.10.057. 29107418

[ref48] Hazlehurst A.; Tiffin L.; Sumner M.; Taylor M. Quantification of microfiber release from textiles during domestic laundering. Environmental Science and Pollution Research 2023, 30, 43932 10.1007/s11356-023-25246-8. 36680713 PMC10076413

[ref49] Hernandez E.; Nowack B.; Mitrano D. Polyester Textiles as a Source of Microplastics from Households: A Mechanistic Study to Understand Microfiber Release During Washing. Environ. Sci. Technol. 2017, 51 (12), 7036–7046. 10.1021/acs.est.7b01750. 28537711

[ref50] De Falco F.; Di Pace E.; Cocca M.; Avella M. The contribution of washing processes of synthetic clothes to microplastic pollution. Sci. Rep. 2019, 9, 6633 10.1038/s41598-019-43023-x. 31036862 PMC6488573

[ref51] Volgare M.; De Falco F.; Avolio R.; Castaldo R.; Errico M.; Gentile G.; et al. Washing load influences the microplastic release from polyester fabrics by affecting wettability and mechanical stress. Sci. Rep. 2021, 11 (1), 1–12. 10.1038/s41598-021-98836-6. 34593897 PMC8484352

[ref52] Cotton L.; Hayward A. S.; Lant N. J.; Blackburn R. S. Improved garment longevity and reduced microfiber release are important sustainability benefits of laundering in colder and quicker washing machine cycles. Dyes Pigm. 2020, 177, 108120 10.1016/j.dyepig.2019.108120.

[ref53] Jiang L.; Yin M.; Tang Y.; Dai R.; Mo L.; Yang W.; Liang Y.; Huang K. Microfibers shed from synthetic textiles during laundry: Flow to wastewater treatment plants or release to receiving waters through storm drains?. Process Safety and Environmental Protection 2022, 168, 689–697. 10.1016/j.psep.2022.10.039.

[ref54] Choi S.; Kim J.; Kwon M. The Effect of the Physical and Chemical Properties of Synthetic Fabrics on the Release of Microplastics during Washing and Drying. Polymers 2022, 14 (16), 3384 10.3390/polym14163384. 36015640 PMC9412705

[ref55] Kärkkäinen N.; Sillanpää M. Quantification of different microplastic fibers discharged from textiles in machine wash and tumble drying. Environmental Science and Pollution Research 2021, 28 (13), 16253–16263. 10.1007/s11356-020-11988-2. 33340055 PMC7969573

[ref56] Browne M. A.; Ros M.; Johnston E. L. Pore-size and polymer affect the ability of filters for washing-machines to reduce domestic emissions of fibers to sewage. PLoS One 2020, 15 (6), e0234248 10.1371/journal.pone.0234248. 32559201 PMC7304565

[ref57] De Falco F.; Di Pace E.; Avella M.; Gentile G.; Errico M.; Krzan A.; ElKhiar H.; Zupan M.; Cocca M. Development and Performance Evaluation of a Filtration System for Washing Machines to Reduce Microfiber Release in Wastewater. Water, Air, & Soil Pollution 2021, 232 (10), 406 10.1007/s11270-021-05342-6.

[ref58] Napper I. E.; Barrett A. C.; Thompson R. C. The efficiency of devices intended to reduce microfiber release during clothes washing. Sci. Total Environ. 2020, 738, 140412 10.1016/j.scitotenv.2020.140412. 32682545

[ref59] Belzagui F.; Crespi M.; Alvarez A.; Gutierrez-Bouzan C.; Vilaseca M. Microplastics’ emissions: Microfibers’ detachment from textile garments. Environ. Pollut. 2019, 248, 1028–1035. 10.1016/j.envpol.2019.02.059. 31091635

[ref60] Zambrano M.; Pawlak J.; Daystar J.; Ankeny M.; Venditti R. Impact of dyes and finishes on the microfibers released on the laundering of cotton knitted fabrics. Environ. Pollut. 2021, 272 (10), 115998 10.1016/j.envpol.2020.115998. 33199065

[ref61] Ramasamy R.; Subramanian R. B. Enzyme hydrolysis of polyester knitted fabric: A method to control the microfiber shedding from synthetic textile. Environmental Science and Pollution Research 2022, 29 (54), 81265–81278. 10.1007/s11356-022-21467-5. 35729395

[ref62] Ramasamy R.; Subramanian R. B. Microfiber mitigation from synthetic textiles - impact of combined surface modification and finishing process. Environmental Science and Pollution Research 2023, 30 (17), 49136–49149. 10.1007/s11356-023-25611-7. 36773261

[ref63] Raja Balasaraswathi S.; Rathinamoorthy R. Effect of fabric properties on microfiber shedding from synthetic textiles. Journal of the Textile Institute 2022, 113, 789 10.1080/00405000.2021.1906038.

[ref64] Zambrano M.; Pawlak J.; Daystar J.; Ankeny M.; Cheng J.; Venditti R. Microfibers generated from the laundering of cotton, rayon and polyester based fabrics and their aquatic biodegradation. Mar. Pollut. Bull. 2019, 142, 394–407. 10.1016/j.marpolbul.2019.02.062. 31232317

[ref65] Dalla Fontana G.; Mossotti R.; Montarsolo A. Influence of Sewing on Microplastic Release from Textiles During Washing. Water Air and Soil Pollution 2021, 232 (2), 50 10.1007/s11270-021-04995-7.

[ref66] Frost H.; Zambrano M.; Leonas K.; Pawlak J.; Venditti R. Do Recycled Cotton or Polyester Fibers Influence the Shedding Propensity of Fabrics during Laundering?. AATCC Journal of Research 2020, 7 (1), 32–41. 10.14504/ajr.7.S1.4.

[ref67] Cai Y. P.; Mitrano D. M.; Heuberger M.; Hufenus R.; Nowack B. The origin of microplastic fiber in polyester textiles: The textile production process matters. Journal of Cleaner Production 2020, 267 (12), 121970 10.1016/j.jclepro.2020.121970.

[ref68] Jönsson C.; Levenstam Arturin O.; Hanning A.; Landin R.; Holmström E.; Roos S. Microplastics Shedding from Textiles—Developing Analytical Method for Measurement of Shed Material Representing Release during Domestic Washing. Sustainability 2018, 10 (7), 2457 10.3390/su10072457.

[ref69] Rathinamoorthy R.; Raja Balasaraswathi S. Investigations on the impact of handwash and laundry softener on microfiber shedding from polyester textiles. Journal of The Textile Institute 2022, 113, 1428–1437. 10.1080/00405000.2021.1929709.

[ref70] Opperskalski S.; Franz A.; Pantanè A.; Siew S.; Tan E. Preferred Fiber & Materials Market Report, 2022. https://textileexchange.org/knowledge-center/reports/materials-market-report/ (accessed 2023–04–22).

[ref71] British Standards Institute. 2023 Textiles and textile products. Microplastics from textile sources. Determination of material loss from fabrics during washing, ISO 4484-1; BSI Standards Limited: London, 2023.

[ref72] First Sentier MUFG Sustainable Investment Institute. Microfibers: the invisible pollution from textiles, 2022. https://www.firstsentier-mufg-sustainability.com/content/dam/sustainabilityinstitute/assets/research/FSI-Sustainability-Investment-Institute-Report-January2022-final.pdf (accessed 2023–10–10).

[ref73] Kentin E.; Battaglia G. Policies and Perspectives on Regulating Microplastic Fibre Pollution. In Polluting Textiles: The Problem with Microfibers; Weis J., Ed.; Routledge, 2022.

[ref74] Hearle J. W. S. Fibre structure: its formation and relation to performance. In Handbook of Textile Fibre Structure; Woodhead Publishing, 2009; pp 3–21.

[ref75] Lawrence C. Fibre to Yarn. In Textiles and Fashion;Elsevier Ltd., 2015; pp 214–230.

[ref76] Vollrath F.; Porter D.; Dicko C. The structure of silk. Handbook of Textile Fibre Structure 2009, 146–198. 10.1533/9781845697310.1.146.

[ref77] Abdelmeguid A.; Afy-shararah M.; Salonitis K. Investigating the challenges of applying the principles of the circular economy in the fashion industry: A systematic review. Sustainable Production and Consumption 2022, 32, 505–518. 10.1016/j.spc.2022.05.009.

[ref78] Šaravanja A.; Pušić T.; Dekanić T. Microplastics in Wastewater by Washing Polyester Fabrics. Materials 2022, 15, 2683 10.3390/ma15072683. 35408015 PMC9000408

[ref79] APPG on Microplastics (All-Party Parliamentary Group on Microplastics). Microplastic Policies for the Government: First report, 2021. https://www.thewi.org.uk/__data/assets/pdf_file/0003/550038/WI_APPGMicroplastics_Report.pdf (accessed 2022–04–10).

[ref80] Liu J.; Liang J.; Ding J.; Zhang G.; Zeng X.; Yang Q.; Zhu B.; Gao W. Microfiber pollution: an ongoing major environmental issue related to the sustainable development of textile and clothing industry. Environment, Development and Sustainability 2021, 23 (8), 11240–11256. 10.1007/s10668-020-01173-3.

